# How training culture normalizes sport bullying: suffering, obedience, and structural violence in athlete development

**DOI:** 10.3389/fpsyg.2026.1885241

**Published:** 2026-07-27

**Authors:** Huancheng Huang, Yuanyuan Chen, Yu Wan, Lu Jiang, Shulin Sun, Luqi Huang

**Affiliations:** School of Physical Education, Southwest University, Chongqing, China

**Keywords:** culture of obedience, culture of suffering, moral disengagement, safe sport, sports bullying, structural violence

## Abstract

Sports bullying is increasingly recognized as a pervasive public health and athlete welfare crisis, yet the micro-level mechanisms that legitimize and sustain it remain underexplored. This study conceptualizes how entrenched “training cultures” normalize structural violence in competitive sports. Positioned as a critical integrative review and an exploratory multiple-case qualitative study, the research utilizes a three-stage thematic analysis. The empirical corpus comprises secondary archival data from two quintessential incidents (the U.S. Maggie Haney case and the Chinese Fu Jiali case), triangulated with primary data from 12 semi-structured interviews with athletes. The findings reveal that the intersection of the “culture of suffering” (enduring hardship) and the “culture of obedience” operates as a “five-fold cultural obscuration mechanism”–encompassing legitimation, normalization, silencing, internalization, and collusion. This mechanism induces severe psychological consequences, including moral disengagement and learned helplessness, coercing athletes to internalize maltreatment as a developmental necessity while fostering systemic bystander silence. By reframing sports bullying from an interpersonal anomaly to a systemic condition sustained by cultural architectures, this paper provides a theoretical scaffolding to delineate rigorous pedagogical training from structural violence. Ultimately, effective governance requires moving beyond reactive punishments toward proactive cultural reconstruction, cognitive restructuring, and multi-level structural accountability.

## 1 Introduction

Sports are widely celebrated for building character, instilling discipline, and fostering resilience. Central to athletic development are two core values: enduring hardship (often contextualized culturally as “eating bitterness”) and strict obedience to coaching authority. While these traits are essential for athletic success, a critical inflection point emerges when they transcend pedagogical ethics and fossilize into rigid organizational dogma. At this juncture, rather than serving as developmental scaffolding, these cultural values are frequently weaponized to suppress dissent, rationalize harm, and protect hierarchical power structures (Galtung, 1969, 1990; Ríos et al., 2022; Vveinhardt and Fominiene, 2019, 2020; Warner and Knoester, 2025; Rook et al., 2023; Srivastava et al., 2023; Stefaniuk and Bridel, 2018; Vveinhardt et al., 2019, 2020).

The prevalence of abuse in athletic environments is well-documented. The International Olympic Committee (IOC) estimates that 30%–70% of athletes experience some form of harassment or abuse during their careers, establishing sports bullying as a critical global crisis (Mountjoy et al., 2016). Previous research has effectively documented the spatial dynamics, demographic vulnerabilities, and long-term psychological trauma associated with bullying, emotional abuse, and toxic coaching practices (Kalina et al., 2025; Pilkington et al., 2024; Warner and Knoester, 2025; Zhang et al., 2026; Wang et al., 2024; Xiang et al., 2024; Ball et al., 2021; Banjac et al., 2020; Collot-D’Escury and Dudink, 2010; Denison et al., 2020; Evans et al., 2016; Flores et al., 2021; Fisher and Dzikus, 2017; Jewett et al., 2020; Keegan et al., 2010; Herrick and Duncan, 2020; Karafil and Pehlivan, 2025; Mishna et al., 2019; Newman et al., 2021; Nery et al., 2020; Fisher and Dzikus, 2017; Srivastava et al., 2023). Extant literature confirms that immense competitive pressures and strict institutional hierarchies create environments highly susceptible to interpersonal transgressions.

However, current literature predominantly focuses on identifying risk factors and detailing subsequent trauma, leaving a crucial micro-level question under-explored: Why are these abusive behaviors systematically perceived by both perpetrators and victims as acceptable, necessary, or even legitimate? This theoretical gap is significant. Without understanding the legitimation mechanisms of harm, systemic abuses are frequently dismissed as mere interpersonal conflicts, communication breakdowns, or “tough coaching.” When the cultural architecture of sports inherently shields abuse under the guise of “building character” or “inevitable collective sacrifice,” traditional punitive measures against individual coaches fail to dismantle the root causes of the violence. Consequently, the prolonged persistence of sports bullying is sustained not merely by its clandestine nature, but by its continuous discursive reconstruction as an integral component of athletic training.

To address this limitation, this study necessitates a paradigm shift in sports bullying research–moving from the mere identification of risk factors to the rigorous analysis of cultural legitimation mechanisms. Specifically, we investigate how the intertwined cultures of “suffering” and “obedience” operate to normalize structural violence. This paper articulates three progressive inquiries:

Through what specific mechanisms do the cultures of “suffering” and “obedience” obscure the reality of bullying?

How is this obscuration institutionalized and reproduced within organizational practices?

How can sports governance pivot from retrospective punitive measures toward proactive cultural reconstruction and structural accountability?

By integrating a critical literature review with qualitative multiple-case analyses and semi-structured interviews, this study bridges theories of structural violence, moral injury, and psychological safety to conceptualize a “five-fold cultural obscuration mechanism model.” The scholarly contribution of this paper lies in its definitional recalibration: it posits that sports bullying functions not as a marginal anomaly, but as a constitutive condition actively sustained by specific sporting cultures. Recognizing this dynamic substantially deepens the current understanding of athlete abuse, underscoring that effective governance pathways must target systemic, cultural, and power architectures rather than isolating individual perpetrators.

## 2 Materials and methods

### 2.1 Study design

This study employed a qualitative, analogical research design, utilizing semi-structured interviews and secondary archival data as the primary analytical corpus. A critical integrative review was initially conducted to synthesize extant research gaps and formulate preliminary theoretical constructs. Subsequently, multiple-case analyses were utilized to empirically verify, calibrate, and refine these constructs; furthermore, through semi-structured interviews with athletes (including both active and retired) across various sports, the authors ultimately deduced and substantiated the “five-fold cultural obscuration mechanism” model. Acknowledging the highly clandestine nature of sports bullying, acquiring primary interview data directly from traumatized cohorts presents formidable ethical and methodological impediments.

### 2.2 Data collection and case selection

A theoretical sampling methodology was adopted. Semi-structured interviews were employed, utilizing the interviewees’ firsthand accounts and conversational analysis to demonstrate the applicability of the model. The cases and interviewees must possess robust typicality and representativeness, effectively illustrating the operational logic of cultural obscuration mechanisms across disparate institutional paradigms; furthermore, the incidents must feature a high degree of public transparency, encompassing comprehensive multi-actor narratives. Two quintessential cases were ultimately selected:

The Maggie Haney Gymnastics Abuse Case (USA): Exemplifying the morphology of bullying within an elite sporting architecture.

The Fu Jiali Gymnastics Fall Case (China): Illustrating the morphology of bullying within the youth training infrastructure.

To ensure analytical rigor, a comprehensive data corpus was systematically constructed, comprising both primary interview data and secondary archival documents:

Primary Interview Data (*N* = [12] interviewees): Semi-structured interviews were conducted with [12] athletes (comprising [4] active and [8] retired athletes) across various sports to validate the structural mechanisms.

Secondary Archival Data (*N* = [15] documents): The data collection spanned the specific evolutionary periods of each case. The Maggie Haney case data covered [6, 2019] to [4, 2020], while the Fu Jiali case data encompassed [11, 2025] to [2, 2026].

The distribution of secondary sources across the cases is as follows:

The Maggie Haney Case: [5] authoritative media articles The Fu Jiali Case: [10] in-depth reports from state-accredited media.

Inclusion and Exclusion Criteria: Documents were included if they provided primary factual accounts, direct quotes from stakeholders (victims, coaches, officials), or organizational policy responses regarding the incidents. Documents were excluded if they were mere opinion pieces, unverified social media speculations, or repetitive syndicated publications lacking novel information. Source Credibility Evaluation: To ensure credibility, source triangulation was strictly enforced. Claims originating from media reports were cross-referenced against legal and official organizational communiqués. Documents that failed to corroborate fundamental factual timelines were excluded from the primary analytical corpus.

### 2.3 Analytical procedure and coding strategy

The study employed a systematic thematic analysis approach to process the qualitative data (Braun and Clarke, 2006). To ensure a transparent analytical pathway from empirical material to the final theoretical model, a three-stage coding procedure was operationalized, facilitated by

Open Coding (Generating Initial Categories): In the first phase, all selected documents were read longitudinally. Meaningful text segments pertaining to organizational responses, power dynamics, and stakeholder behaviors were line-by-line coded. For example, textual descriptions of coaches isolating athletes were initially coded with descriptive labels such as “restricting communication” or “information blockade.”

Axial Coding (Identifying Themes): In the second phase, initial open codes were iteratively compared and grouped into broader conceptual categories. By analyzing the relationships between these categories and referencing Galtung’s structural violence paradigm, we identified distinct phases of systemic cover-ups. For instance, codes like “information blockade” and “confiscating devices” were merged into the broader theme of “Information Segregation.”

Selective Coding (Theoretical Model Emergence): Finally, the core themes identified in the previous step were sequenced chronologically and logically to explain the overarching phenomenon. Through cross-case synthesis, comparing the U.S. model with the Chinese model, the contextual variances were stripped away to reveal a universal underlying structure. This rigorous abstraction process directly yielded the five-fold cultural obscuration mechanism model, transforming raw narrative data into a structured theoretical framework.

### 2.4 Limitations of secondary data and mitigation strategies

The reliance on publicly available secondary data inherently risks media narrative bias and informational fragmentation. To mitigate these methodological vulnerabilities, the study employed a “triangulation” strategy. Reports detailing identical events from initial publishing outlets were cross-referenced against subsequent official communiqués, legal testimonies, and public declarations by involved parties. Highly subjective or emotive lexicon was systematically excised, extracting exclusively objective factual delineations concerning “behavioral occurrences,” “organizational responses,” and “power dynamics,” thereby ensuring the analytical corpus’s objectivity and reliability.

## 3 Results: case background and event review

### 3.1 The US Maggie Haney Gymnastics Abuse Case

Maggie Haney, a prominent American gymnastics coach who developed numerous Olympic and World medalists, was subjected to a 2-years investigation by USA Gymnastics. In 2020, the organization concluded that Haney had perpetrated systemic abuse against at least ten minor athletes between 2013 and 2019, ultimately imposing an 8-years suspension.

Haney’s abusive repertoire predominantly encompassed:

Physical Abuse: Coercing athletes to train through severe trauma, including fractures and ligamentous tears, inflicting permanent physiological damage on multiple athletes.

Verbal and Emotional Abuse: Denigrating menstruating athletes as “too fat” and “disgusting,” utilizing derogatory rhetoric to systematically erode athletes’ self-worth and psychological integrity.

Behavioral Control: Engaging in physical intimidation (e.g., hair-pulling, shoving), barring parental access to training facilities, and invasively monitoring athletes’ dietary and social engagements.

Collective Punishment: Retaliating against individual grievances by subjecting the entire cohort to punitive, high-intensity training, thereby coercing peers into isolating the complainant.

Despite the ensuing public outcry and the resignation of several USA Gymnastics executives, a contingent of coaches and parents continued to advocate for Haney, rationalizing her “draconian demands” as an indispensable catalyst for cultivating elite athletic performance.

### 3.2 The Chinese Fu Jiali Gymnastics Fall Case

Fu Jiali, a former Group A all-around gymnastics champion at the Zhejiang Provincial Games, was designated as a highly coveted reserve talent within the provincial athletic infrastructure. In November 2025, driven to desperation by protracted corporal punishment, verbal intimidation, and coercive extortion perpetrated by head coach Tang Yan and assistant coach Shi Jia, 13-years-old Fu Jiali leapt from the fourth floor of the Zhejiang College of Sports training facility, sustaining catastrophic organ ruptures.

According to familial testimonies and institutional insiders, the systemic abuse spanned nearly 2 years:

Corporal Punishment and Violence: Flagellating athletes’ extremities and backs with gymnastic apparatuses, and mandating prolonged stationary exposure under extreme solar heat.

Verbal Intimidation: Employing coercive threats, such as, “If you exhibit insubordination, abandon any aspirations of championship,” and “Disclosure to your parents will result in severe physical retribution.”

Coercive Extortion: Extorting material gifts and financial subsidies from parents, weaponizing the allocation of training time and competitive opportunities as leverage.

Psychological Control: Orchestrating the systematic isolation of Fu Jiali by prohibiting peer interaction, thereby inducing profound psychological terror and alienation.

In the immediate aftermath, institutional administrators obfuscated investigations by withholding surveillance records, delaying medical disbursements, and officially disassociating the institution from the “personal act” of the fall. It was solely the escalation of public outrage that compelled the Zhejiang Provincial Sports Bureau to instigate a formal inquiry and suspend the implicated personnel.

## 4 Theoretical framework

To rigorously dissect the aforementioned mechanisms, this study operationalizes Johan Galtung’s theory of structural violence and Michel Foucault’s conceptualization of disciplinary power as its core theoretical scaffolding.

Crucially, this manuscript explicitly avoids cultural determinism and does not imply that any specific national or cultural tradition inherently produces abuse. Values such as endurance (eating bitterness), discipline, and respect for authority are inherently neutral and can be profoundly developmentally meaningful in sport. This study does not critique their pedagogically sound manifestations. Rather, it interrogates their alienated iterations. A more balanced interpretation frames these cultural values as potential risk amplifiers rather than direct causes of abuse. They become harmful only when they are weaponized to rationalize coercion, silence, humiliation, or violence under certain institutional conditions.

Primarily, this manuscript does not critique the pedagogically sound manifestations of “eating bitterness” and “obedience” (i.e., calibrated training loads and normative discipline conducive to character development). Rather, it interrogates their alienated iterations–when “eating bitterness” systematically violates human dignity, and “obedience” systematically expropriates fundamental human rights. Utilizing Galtung’s (1990) “Violence Triangle” paradigm, this study posits that direct violence (e.g., physical battery, verbal assault) within sporting arenas proliferates unchecked because it is ideologically legitimized by cultural violence (the alienated narratives of endurance and submissiveness). This legitimation is subsequently ossified by structural violence (the coach’s hegemonic control over resources and the insularity of the institutional system), thereby ensuring its continuous reproduction.

Furthermore, Foucault’s theory of “Disciplinary Power” provides a granular, micro-sociological explication for the culture of obedience. Sports training infrastructures (e.g., isolated academies) operate spatially analogous to the “panopticon.” Through the meticulous micro-segmentation of athletes’ anatomies, the draconian regimentation of time, and pervasive hierarchical surveillance, “docile bodies” are systematically manufactured. Within this panoptic power matrix, obedience is internalized as a somatic instinct, while any nascent consciousness of critical inquiry or resistance is proactively dissolved by systemic disciplinary mechanisms.

### 4.1 The cultural obscuration mechanism model

Synthesizing these theoretical frameworks, this study postulates the “Five-fold Cultural Obscuration Mechanism Model” regarding sports bullying (see Table 1). The model hypothesizes that the cultures of “eating bitterness” and obedience legitimize deleterious behaviors and perpetuate their reproduction through five interrelated, mutually reinforcing phases:

**TABLE 1:** Cultural obscuration mechanism model and its psychological implications.

Mechanism	Organizational manifestation	Psychological process	Consequential impact
Legitimation	Harm is discursively framed as training, discipline, tradition, or collective necessity.	Harm is recontextualized as an “indispensable cost.”	Recognition of abuse is significantly diminished.
Normalization	Repeated occurrence facilitates integration into institutional routine.	Tolerance thresholds elevate; emotional/cognitive sensitivity decays.	Exceptional harm morphs into perceived “normality.”
Silencing	Absence of robust reporting channels combined with high socio-occupational costs of expression.	Help-seeking behaviors atrophy; expression is actively avoided.	Systemic abuses remain operationally invisible.
Internalization	Victims attribute traumatic experiences to perceived personal inadequacy.	Induction of self-blame, profound shame, and learned helplessness.	Catastrophic decline in victim self-efficacy.
Collusion	Bystander apathy, managerial acquiescence, and deliberate institutional delay.	Widespread diffusion of responsibility and moral disengagement.	Uninterrupted institutional reproduction of bullying.

Legitimation: The discursive reinterpretation of harm as normative training, strict discipline, venerated tradition, or collective necessity.

Normalization: The transmutation of exceptional, severe harm into ubiquitous daily praxis through persistent repetition and institutional routine.

Silencing: The systemic suppression of grievances, dissent, and whistleblowing via exacerbated power asymmetries and the elevated socio-occupational costs of expression.

Internalization: The psychological coercion of victims to misattribute institutional harm to personal deficits, inherent flaws, or psychological fragility.

Collusion: The establishment of collective systemic maintenance for bullying, facilitated by bystander apathy, managerial complicity, and institutional obfuscation.

## 5 Analysis of core mechanisms

The five constituent elements of the cultural obscuration mechanism do not operate in a vacuum; rather, they constitute a synergistic, dynamic closed-loop. Legitimation confers initial moral validity upon harm; normalization degrades sensitivity to abuse through pervasive repetition; silencing precludes the exposure of harm via coercive power dynamics; internalization mandates victim self-condemnation; and collusion guarantees the uninterrupted reproduction of harm through collective complicity.

### 5.1 Legitimation mechanism: moral justification and discursive reframing

The primary utility of the “eating bitterness” culture resides in its provision of a legitimizing lexicon. Viewed through the prism of social psychology, this represents a quintessential strategy of “moral justification” within the framework of moral disengagement (Bandura, 1999; Festinger, 1957). Perpetrators and administrators systematically endow excessive corporal punishment and psychological degradation with exalted social and moral imperatives, packaging them as methodologies for “fortifying resilience,” “securing collective prestige,” or operating under the maxim that “draconian pedagogy yields unparalleled excellence.”

In the Fu Jiali tragedy, the coaching staff rhetorically sanitized chronic physical abuse as “tempering willpower to secure championships,” insisting that “contemporary severity is a prerequisite for future prosperity.” Similarly, Haney’s apologists postulated that “gymnastics is inherently brutal; Olympic gold is inaccessible absent the endurance of profound suffering.” Beyond direct moral justification, perpetrators frequently deploy ancillary disengagement strategies, including the “displacement of responsibility” (“I was merely executing organizational directives”) and “dehumanization” (“Athletes are engineered to endure; they cannot exhibit the fragility of ordinary civilians”), thereby systematically degrading the moral reprehensibility of their actions.

### 5.2 Normalization mechanism: habituation and systemic desensitization

When traumatic stimuli are relentlessly administered, they shed their status as anomalous “events” and transmute into institutionalized norms. The behavioral science paradigm of habituation elucidates this trajectory: prolonged, repetitive exposure to specific stimuli (e.g., bullying) precipitously diminishes an individual’s emotional and cognitive reactivity.

In the Haney case, veteran-led bullying rituals, sustained over months, were normalized as “mandatory rites of passage.” Novice athletes were immediately subjected to verbal vitriol and systemic servitude by senior peers, a dynamic the coaching staff deliberately ignored. In the Fu Jiali incident, physical battery, forced static endurance, and caloric deprivation devolved into quotidian training protocols. Consequently, athletes progressively internalized the paradigm that “coercive physical punishment is a standard pedagogical tool.” This normalization engenders collective psychological desensitization; the trauma is recalibrated by organizational constituents (crucially including the victims) as the baseline equilibrium of the training environment, entirely stripping it of its “aberrant” classification.

### 5.3 Silencing mechanism: power distance and expressive suppression

The culture of obedience engineers profound power asymmetries within sporting architectures. In systems heavily predicated on coaches for the dispensation of critical resources (e.g., competition rosters, career progression), challenging authority incurs exorbitant socio-professional penalties. Silencing, therefore, is not symptomatic of a victim’s cognitive deficit, but rather a hyper-rational avoidance strategy dictated by professional survival.

Foucault’s conceptualization of disciplinary power further exposes the structural scaffolding of this silence: the corporeal discipline inherent to athletic training (e.g., draconian scheduling, rigid biomechanical standardization), spatial discipline (e.g., sequestered training complexes, isolated dormitories), and discursive discipline (e.g., the dogma that “unquestioning obedience is an athlete’s supreme duty”) collaboratively engineer absolute submissiveness. In this matrix, silence solidifies into a subconscious behavioral reflex. Fu Jiali had repeatedly signaled her distress to her parents, yet the pervasive fear that antagonizing the coach would annihilate her career trajectory mandated parental silence. Similarly, peer athletes were paralyzed by the threat of retaliatory ostracism, ensuring an impenetrable veil of secrecy.

### 5.4 Internalization mechanism: learned helplessness and maladaptive self-attribution

The most insidious obscuration achieved by systemic bullying is the cognitive indoctrination of the victim to believe the abuse is “warranted.” Athletes ensnared in protracted, inescapable environments of oppression exhibit a profound susceptibility to learned helplessness. Here, the cultures of hardship and obedience achieve devastating resonance: realizing that no magnitude of exertion can mitigate their humiliation, individuals cease resistance and maladaptively attribute their trauma to internal, stable, and pervasive personal flaws.

Fu Jiali’s final correspondence poignantly captured this internalized destruction: “It is my inherent inadequacy; I continuously fail my routines, thereby inciting the coach’s justifiable wrath.” An analogous sentiment was echoed by a victim in the Haney investigation: “I genuinely believed the coach’s verbal and physical assaults were merely the consequence of my own stupidity and insufficient dedication.” This internalization mechanism annihilates the athlete’s self-efficacy, transmuting external structural violence into debilitating internal psychological attrition, frequently precipitating catastrophic outcomes such as clinical depression and suicidality (Hawkins, 2026; Kavanagh et al., 2017; Chmielewski et al., 2024; Zou et al., 2018).

### 5.5 Collusion mechanism: diffusion of responsibility and the institutional bystander effect

Sports bullying is perpetuated not solely by the primary aggressors, but by the pervasive complicity of the organizational apparatus. In the insulated ecosystems of sports teams or academies, the passive silence of peers and the systemic obfuscation by administrators precipitate a textbook diffusion of responsibility. When pervasive abuse is universally witnessed, a cognitive diffusion occurs: individuals assume intervention is the purview of others, or logically deduce that managerial acquiescence denotes the behavior’s inherent validity.

In the Fu Jiali case, institutional leadership possessed explicit cognizance of the abusive methodologies yet opted for calculated acquiescence to preserve “squad stability” and ensure “competitive medal yields.” Teammates maintained silence to evade social excommunication (Asch, 1956), and disturbingly, a subset of parents actively opposed punitive measures against the coaches, tethered to the belief that “severity breeds excellence.” This ubiquitous collusion guarantees that bullying transcends isolated deviance, establishing itself instead as an organizational imperative tacitly sanctioned by the collective majority.

To systematically demonstrate the link between the empirical case evidence and the theoretical claims of the proposed “cultural obscuration mechanism model,” Table 1 delineates the specific pathways through which structural violence is legitimized and perpetuated. By mapping the key maltreatment behaviors against their underlying cultural justifications and subsequent psychological/behavioral outcomes, the table elucidates how both market-driven (Haney case) and state-sponsored (Fu case) athletic architectures utilize distinct cultural narratives to achieve similar outcomes of systemic obscuration.

**Table:** 

Case Context	Key Bullying, Abuse, or Maltreatment Behaviors	Cultural Justifications for Legitimizing Behaviors	Psychological Mechanisms Involved	Behavioral Outcomes	Consequences for Athlete Well-being
The Maggie Haney Case (USA) (Market-driven, elite sporting architecture)	Physical & Verbal Abuse: Coercing training through severe trauma (fractures); body-shaming (“too fat”). Control: Physical intimidation (shoving, hair-pulling); barring parental access. Collective Punishment: Punishing the cohort for individual grievances.	“Tough Coaching” Paradigm: Rationalizing draconian demands and abuse as an “indispensable catalyst” for cultivating Olympic/World medalists. Performance Supremacy: Viewing psychological degradation as necessary for building “competitive toughness.”	Moral Disengagement: Parents and officials justifying the abuse due to the production of medals. Conformity: Peers conforming to the coach’s demands to isolate complainants out of fear of collective punishment.	Delayed Reporting: Systemic abuse remained hidden and unchallenged from 2013 to 2019. Bystander Inaction: A contingent of coaches and parents actively defended the perpetrator post-exposure. Compliance & Silence: Athletes submitting to dangerous training regimens.	Physical: Permanent physiological damage (fractures, ligamentous tears). Psychological: Systematic erosion of self-worth, emotional exhaustion, and compromised psychological integrity.
The Fu Jiali Case (China) (State-sponsored youth training infrastructure)	Corporal Punishment: Flagellation with apparatuses; prolonged exposure to extreme heat. Intimidation & Control: Threats of ending athletic careers; violent retribution for disclosing abuse to parents; enforced peer isolation. Extortion: Coercing financial subsidies and gifts from parents.	Absolute Authority & Obedience: Utilizing the hierarchical state system (Juguo tizhi) where coaches control resource and career allocation (“If you exhibit insubordination, abandon any aspirations”). “Eating Bitterness”: Normalizing severe corporal punishment and deprivation as traditional disciplinary pedagogy.	Learned Helplessness: The victim’s realization that reporting internally leads to retaliation, inducing profound despair. Authority Obedience: Submitting to extortion and physical violence due to systemic power imbalances.	Silence: Victim concealing abuse from parents due to coercive threats. Institutional Obfuscation: Administrators withholding surveillance and dismissing trauma as a “personal act.” Extreme Withdrawal: The victim leaping from a building as a desperate escape.	Physical: Catastrophic organ ruptures requiring extensive medical intervention. Psychological: Profound psychological terror, extreme alienation, and absolute desperation culminating in a suicide attempt.

## 6 Divergent obscuration logics across sporting typologies

The obscuration of sports bullying does not manifest as a monolithic entity; rather, it exhibits highly nuanced, context-dependent variations across disparate sporting arenas.

### 6.1 Youth sport

Within youth demographics, systemic harm is frequently inextricably intertwined with normative educational, pedagogical, and socialization processes. Given their chronological immaturity, severely constrained bargaining leverage, and the punitive costs of system exit, young athletes are disproportionately predisposed to interpret abusive treatment as legitimate pedagogy. For example, within a provincial Chinese swimming academy’s “veteran-mentors-rookie” tradition, senior athletes inflicted protracted verbal degradation, physical intimidation, and forced labor upon junior cohorts. The deliberate abstention of intervention by coaches and institutional oversight culminated in a 12-years-old athlete being diagnosed with severe clinical depression and anxiety. Horrifically, the team’s internal narrative legitimized these abuses as essential “team bonding” and “courage fortification,” aligning seamlessly with the obscuration logic of the “eating bitterness” paradigm.

### 6.2 Elite sport

In elite echelons, abuse is intricately enmeshed with acute performance imperatives and the construction of professional identity. Junior athletes transitioning into elite architectures must expeditiously assimilate into novel cultural paradigms and relational hierarchies while simultaneously sustaining peak performance under intense public scrutiny and rigorous lifestyle policing. Illustratively, a prominent Chinese national table tennis player who dared to question a coach’s methodological efficacy was subjected to a punitive 2-years competitive embargo, effectively severing their access to international arenas. The coach publicly validated this draconian exile as a necessary measure for “cultivating absolute obedience” and “safeguarding team cohesion,” while peers, terrified of mirroring this professional decapitation, maintained absolute silence.

### 6.3 Team sports

Within team sports, bullying demonstrates a pronounced propensity to metastasize within specific spatial architectures. Locker rooms, sequestered training camps, transit vehicles, and internal debriefing sanctums–characterized by negligible external surveillance and hyper-dense internal interaction–emerge as primary incubators for systemic abuse. For instance, within the locker room of a professional Chinese football club, veteran personnel subjected rookies to profoundly degrading “initiation rites,” encompassing coerced alcohol consumption, forced subjugation, and menial labor. Club executives implicitly sanctioned these rituals under the facade of “forging robust team cohesion,” thereby inflicting profound psychological and physiological trauma upon the incoming athletes.

## 7 Discussion

### 7.1 Theoretical contributions to the discourse on sports bullying

The central thesis of this manuscript is that sports bullying must not be trivialized as a technical debate over “pedagogical severity”; it must be rigorously interrogated as a normative crisis concerning “the permissible parameters of institutional harm.” This study substantially advances the extant scholarly discourse across three primary dimensions:

Paradigmatic Rupture: By transcending the conventional “risk factor–individual consequence” analytical binary, this research introduces a robust “legitimation mechanism” framework, thereby elevating sports bullying from an isolated behavioral aberration to a manifestation of systemic structural violence.

Elevation of Explanatory Variables: The study promotes the “culture of eating bitterness” and the “culture of obedience” from passive contextual background variables to active, core explanatory drivers, thereby elucidating the dynamic agency of culture in the continuous reproduction of harm.

Comprehensive Mechanistic Explication: The introduction of the “collusion mechanism” successfully resolves a critical gap in the literature regarding why bullying persists in highly visible environments, addressing the historical neglect of group-level socio-psychological mechanisms.

### 7.2 Interfacing with global research agendas

A pervasive and enduring controversy within international sports bullying research is the semantic and practical difficulty of delineating the boundary between rigorous training and definitive abuse. The five-fold mechanism model delineated herein provides highly operationalizable criteria for establishing this demarcation. If a specific behavior contemporaneously exhibits the following five characteristics, it has unequivocally breached the threshold of pedagogical rigor and constitutes bullying:

It is discursively legitimized as an “indispensable pedagogical strategy” or an “inevitable developmental phase.”

It has calcified into daily organizational routine, perceived as standard by the constituent majority.

Victims are coerced into silence regarding grievances due to acute fear of professional or social retaliation.

Victims exhibit cognitive distortion, attributing the perpetrated abuse to their own inherent inadequacies.

Organizational administrators and bystanders consistently exhibit passive silence or active acquiescence.

These empirical markers furnish sports organizations with an unambiguous framework for the rapid identification and decisive intervention of abusive praxes.

### 7.3 Governance imperatives: cultural reconstruction and structural accountability

To enact meaningful mitigation of sports bullying, governance paradigms must decisively pivot from reactive, ex-post punitive models toward proactive cultural reconstruction and uncompromising structural accountability. Drawing upon the findings of the proposed cultural obscuration mechanism model and aligning with contemporary Safe Sport literature, it is imperative to dismantle the deeply entrenched cultures of “suffering” and “obedience” through a comprehensive, multi-level safeguarding framework:

Individual Level (Empowerment and Cognitive Restructuring): To counteract the cognitive distortion and learned helplessness induced by the “culture of suffering,” interventions must focus on athlete empowerment. Sports programs must systematically integrate athlete rights education and mental health literacy into standard curricula. Specifically, athletes must be trained in psychological boundary recognition–enabling them to distinguish between rigorous pedagogical demands and abusive practices–and be equipped with concrete help-seeking skills to break the internalized cycle of compliance.

Interpersonal Level (Relational Dynamics and Bystander Action): Addressing the “culture of obedience” requires reshaping the immediate micro-social environment. Coach education must be fundamentally reformed to prioritize autonomy-supportive coaching over dictatorial control, eliminating the rationalization of abuse as “tough coaching.” Furthermore, implementing trauma-informed communication protocols and mandating bystander intervention training for peers, parents, and assistant coaches are critical to dismantling the passive silence and group conformity that perpetuate structural violence.

Organizational Level (Institutional Integrity and Reporting): To prevent institutional obfuscation and the weaponization of managerial power, sports clubs and academies must enact stringent structural safeguards. This includes the appointment of independent safeguarding officers and the establishment of limits on unilateral coach authority (e.g., separating coaching duties from resource allocation and medical decisions). Organizations must operationalize highly secure, confidential reporting systems and enforce strict policies guaranteeing protection from retaliation, thereby ensuring victims and whistleblowers are not subjected to collective punishment or career sabotage.

Policy Level (National Frameworks and Legal Accountability): Macro-level governance must transcend organizational self-regulation. National sports governing bodies must ratify comprehensive national safeguarding frameworks and establish clear legal accountability by integrating an “Athlete Rights Protection” charter into national Sports Laws. Crucially, this level requires transparent disciplinary procedures and independent monitoring. Assimilating international praxis, the establishment of autonomous entities–akin to the U.S. Center for SafeSport or the UK Sport Anti-Abuse Unit–is vital. These bodies must possess unfettered investigative and disciplinary jurisdiction, entirely divested from existing sports bureaucracies, to effectively dismantle the deeply entrenched culture of institutional self-preservation.

## 8 Limitations and future trajectories

This investigation acknowledges several methodological limitations: (1) The empirical sample is inherently constrained, focusing on two primary and three supplementary cases; the cross-cultural and cross-disciplinary universality of these conclusions warrants further expansive validation. (2) The analysis lacks primary qualitative insights from the perspectives of perpetrators and institutional managers, precluding a fully dimensional analysis of the multi-actor negotiations underlying the obscuration process. (3) The reliance on secondary archival data precludes a clinical exploration of the victims’ phenomenological experiences of trauma and psychological deterioration.

Future research agendas should prioritize: (1) Empirical Quantification: The development and validation of a “Sports Cultural Obscuration Scale” to facilitate largen quantitative assessments across diverse regional and athletic cohorts. (2) Qualitative Deep-Dives: The execution of phenomenological, in-depth interviews with athletes, coaches, guardians, and administrators to uncover the micro-sociological mechanics of bullying obscuration. (3) Comparative Analytics: The execution of cross-national and cross-disciplinary comparative studies to isolate variables influencing obscuration mechanisms. (4) There is a potential risk of confirmation bias inherent in our case selection. The analyzed cases represent highly visible, extreme instances of abuse. While such extreme or “revelatory” cases are highly appropriate and valuable for exploratory theory building–specifically for rendering invisible structural violence visible–they inherently contain the outcome being investigated. The current study lacks a contrasting case (e.g., a high-performance training environment characterized by strict discipline but devoid of documented abuse). The absence of a non-abusive control case limits our ability to empirically delineate the exact operational boundary between demanding pedagogical rigor and the onset of structural violence. (5) Comparative Case Designs: The execution of comparative studies incorporating non-abusive, high-performance athletic cohorts to serve as contrasting cases. This will be crucial for clarifying the exact tipping point where demanding training mutates into bullying, thereby controlling for confirmation bias.

## 9 Conclusion

Operating at the intersection of organizational culture, power dynamics, and human rights advocacy, this exploratory study interrogates how the venerated cultures of “eating bitterness” (enduring hardship) and obedience function to systematically camouflage the reality of bullying within sporting contexts. The primary conceptual contribution of this manuscript lies in the conceptualization of the “five-fold cultural obscuration mechanism model.” By identifying the synchronized mechanisms of legitimation, normalization, silencing, internalization, and collusion, this study provides a theoretical framework that reframes sports bullying from an isolated interpersonal aberration into a manifestation of systemic structural violence. The analysis suggests that through these intersecting cultural lenses, profoundly deleterious behaviors are discursively laundered into acceptable forms of training, discipline, and tradition, thereby facilitating their uninterrupted institutional reproduction.

Contextualized within the realities of both market-driven elite systems and state-sponsored infrastructures (such as China’s competitive and collegiate sporting apparatus), the alienation of these cultural narratives appears to be amplified by the confluence of insular youth training environments, gold-medal-centric performance evaluations, and paternalistic coaching hegemonies. The proposed five-fold obscuration mechanism consequently offers a highly relevant analytical lens for understanding the pragmatic necessities of governing sports bullying in these localized scenarios.

However, it is crucial to explicitly acknowledge the exploratory nature of this qualitative design. The proposed model serves as a theoretical scaffolding rather than an empirically validated diagnostic tool. Because the findings are drawn from a delimited multiple-case analysis and semi-structured interviews, we do not claim that the model has been systematically validated across all sporting typologies or cultural contexts. Future large-scale quantitative assessments and multi-actor clinical investigations are required to rigorously test, empirically validate, and calibrate the proposed mechanisms.

Despite these methodological boundaries, the theoretical insights presented herein underscore a critical governance imperative. The ethos that modern sporting institutions must actively cultivate is not one where the endurance of profound degradation serves as the price of admission, nor one where the suppression of autonomy is the prerequisite for order. Rather, the imperative is to architect a culture unequivocally anchored in fundamental dignity, non-negotiable boundaries, and athlete subjectivity. Only when sports organizations possess the theoretical awareness and structural fortitude to confront these historically normalized abuses can the institution of sport genuinely reclaim its foundational mandate: to foster holistic human development, cultivate exceptional capabilities, and elevate collective well-being.

## Data Availability

The original contributions presented in this study are included in this article/supplementary material, further inquiries can be directed to the corresponding author.
